# With both eyes open – translational research using eye-tracking combined with performance-based evaluation among people with severe mental illness

**DOI:** 10.1192/j.eurpsy.2021.372

**Published:** 2021-08-13

**Authors:** S. Regev, N. Josman, A. Mendelsohn

**Affiliations:** 1 Occupational Therapy, University of Haifa, Haifa, Israel; 2 Neurobiology, University of Haifa, Haifa, Israel

**Keywords:** real-life, daily activities, efficiency, Executive functions

## Abstract

**Introduction:**

Individuals with severe mental illnesses (SMI) often find it hard to perform daily activities such as grocery shopping, which require intact executive functions. The use of performance-based evaluations is valuable, but lacks the subjects’ point of view during task performance.

**Objectives:**

The aim of the current presentation is to bring together performance-based observation and cognitive science methods to provide insights regarding real-life behavior and problem solving in SMI populations.

**Methods:**

In this quasi-experimental study, forty-three individuals performed the Test of Grocery Shopping Skills (TOGSS) while wearing an eye-tracking device. Eye-movement patterns served as a proxy of executive functions in people with and without SMI during a real-life ingredient selection task. We hypothesized that significant differences will be found between people with SMI and controls in TOGSS sub-outcomes as well as in eye-fixation durations.

**Results:**

TOGSS sub-outcomes indicative of performance efficiency (time and redundancy) were significantly higher in the research group compared to matched controls (P<0.01). Average fixation duration was found to be significantly higher for the research group compared to matched controls (P<0.05) for two of the four item-selection tasks.

**Conclusions:**

These preliminary findings indicate that when confronted with a selection task, individuals with SMI spend more dwelling time while selecting ingredients. Further analyses on these data will examine how this time is spent (e.g. focusing on irrelevant information). The outlined approach may prove beneficial in illuminating specific behavioral and physiological difficulties in individuals with SMI, particularly in the evolving Covid-19 situation, which poses novel social and health-related challenges on real-life tasks.

**Disclosure:**

No significant relationships.

